# Systematic Review and Quality Evaluation of Pharmacoeconomic Studies on Traditional Chinese Medicines

**DOI:** 10.3389/fpubh.2021.706366

**Published:** 2021-08-03

**Authors:** Nan Yang, Huihui Zhang, Taoyi Deng, Jeff Jianfei Guo, Ming Hu

**Affiliations:** ^1^West China School of Pharmacy, Sichuan University, Chengdu, China; ^2^James L. Winkle College of Pharmacy, University of Cincinnati Academic Health Center, Cincinnati, OH, United States

**Keywords:** trad. Chinese medicine, Chinese patent medicines, pharmacoeconomic, system review, quality evaluation

## Abstract

**Objectives:** This study was aimed to find and appraise the available published pharmacoeconomic research on Traditional Chinese Medicine (TCM), to identify related issues and make suggestions for improvement in future research.

**Methods:** After developing a search strategy and establishing inclusion and exclusion criteria, pharmacoeconomic studies on TCM were sourced from seven Chinese and English databases from inception to April 2020. Basic information about the studies and key pharmacoeconomic items of each study were extracted. The quality of each study was evaluated by using the British Medical Journal economic submissions checklist for authors and peer reviewers, focusing on factors such as study design, research time horizon, sample size, perspective, and evaluation methods.

**Results:** A total of 431 published pharmacoeconomic articles with 434 studies on topics including cost-effectiveness, cost-benefit, cost-minimization, cost-utility, or combination analyses were identified and included in this review. Of these, 424 were published in Chinese and 7 in English. These studies conducted economic evaluations of 264 Chinese patent medicines and 70 types of TCM prescriptions for 143 diseases, including those of the central nervous, cardiovascular, respiratory, gynecologyical, and other systems. The studied TCMs included blood-activating agents (such as Xuesaitong tablet, Fufant Danshen tablet, and Danhong Injection), blood circulation promoting agents (such as Shuxuetong injection, Rupixiao tablet, and Fufang Danshen injection), and other therapeutic agents. The overall quality score of the studies was 0.62 (range 0.38 to 0.85). The mean quality score of studies in English was 0.72, which was higher than that of studies in Chinese with 0.62.

**Conclusions:** The quality of pharmacoeconomic studies on TCM was relatively, generally low. Major concerns included study design, inappropriate pharmacoeconomic evaluation, insufficient sample size, or non-scientific assessment. Enhanced methodological training and cooperation, the development of a targeted pharmacoeconomic evaluation guideline, and proposal of a reasonable health outcome index are warranted to improve quality of future studies.

## Introduction

Pharmacoeconomics is a complex science that provides evidences for the optimal allocation and efficient utilization of medical resources. Since the 1970s, it has been increasingly accepted and applied by health care providers, health policy makers, and medical insurance institutions. It has also formed part of the basis of new drug applications, drug pricing, drug purchasing by medical institutions, National Essential Medicines List formulation, medical insurance, and post-marketing drug evaluation (1–3).

With the increasing popularity of and substantial expenditures on complementary and alternative medicine (CAM) therapies globally, the availability of economic evaluations becomes increasingly important (4, 5). However, the quality of those evaluations has significant influence on the popularization and application of the results. A few systematic reviews on economic evaluations of CAM have described findings and quality of available research and have made recommendations for future research (6–10).

Traditional Chinese Medicine (TCM) as one kind of CAM plays an important role in preventing, treating, and curing disorders and diseases (11, 12). Since 1984, the Drug Administration Law of the People's Republic of China has established the policy governing the development both modern medicines and traditional medicines. In 2020, Chinese patent medicine accounted for more than one third of all the drug approvals in China (13) and for 49.07% of all drugs listed in the National Drug Catalog for Basic Medical Insurance of China (14), equaling the proportion of Western medicines. In recent years, with more attention on economic evaluation of medicines, an increasing amount of pharmacoeconomic research has comprehensively evaluated the efficacy, safety and affordability of TCM. This research provides evidence for the rational use of health resources, essential medicine and state medical insurance catalog selection and national drug price negotiations.

The present study aims to find and appraise the available published pharmacoeconomic studies on TCM, analyze the limitations of the research and make suggestions for improved pharmacoeconomic evaluation on TCM in future.

## Materials and Methods

### Data Sources

This systematic review was conducteded in accordance with the Preferred Reporting Items for Systematic Reviews and Meta-Analyses (PRISMA) guidelines. We searched the following databases between their inception (years in parentheses) and April 2020: China National Knowledge Infrastructure(CNKI) (1974), Chongqing VIP Information(VIP) (1989), WanFang Database (1998), Sinomed (1978), Pubmed (1966), Embase (1974), and Cochrane Library (2000).

The search keywords was (“Chinese medicine” OR “Traditional Chinese Medicine” OR “Chinese patent medicine” OR “herbal medicine” OR “natural medicine” OR “Chinese Medicinal Materials” OR “Botanical medicine” OR “Chinese herbal pieces” OR “Chinese medicinal granula”) and (“pharmacoeconomic” OR “economics” OR “Cost Effectiveness Analysis” OR “Cost Utility Analysis” OR “Cost Benefit Analysis” OR “Cost Minimization Analysis” OR “Markov” OR “Decision Tree” OR “Partitioned Survival Model”). Chinese and English terms for search keywords were used when searching the databases. In addition, the references lists of retrieved articles were also searched.

### Study Selection

We included the articles describing pharmacoeconomic evaluation articles on TCM, defined as medicines made according to Chinese medicine theory, such as Chinese patent medicine and Chinese medicine prescription or formula. Articles describing research on traditional Chinese treatment or therapy as interventions such as acupuncture, Guasha or massage or theory papers, reviews, reports, protocols, news, and opinion articles, were excluded. Partial economic evaluations, such as outcome, cost, cost-outcome description, effectiveness (or efficacy) evaluation, and cost analysis, quality of life research, and budget impact analysis were also excluded. Besides, the study country was limited in China and study language was limited in Chinese and English.

### Data Extraction and Quality Assessment

All articles included in the study were read in full and independently judged against inclusion and exclusion criteria of the individual phase by two authors (HH Zhang and TY Deng). Any difference during assessment between the two reviewers was discussed or resolved by a third dependent reviewer (N Yang). A data extraction table was designed using Excel software, and information extracted from each article including title, first author's name and affiliation, journal and year of publication, information about disease, sample size, study design, intervention, and information related cost, health outcomes, uncertainty analysis, perspective, evaluation techniques, and other factors. The data were descriptively analyzed using Microsoft Excel.

The five most representative checklists or guidelines for evaluation of pharmacoeconomic research are the British Medical Journal (BMJ) checklist (15), Quality of Health Economic Studies (QHES) instrument (16), Consensus Health Economic Criteria (CHEC) (17), Philips guideline (18), and Consolidated Health Economic Evaluation Reporting Standards (CHEERS) (19). Among them, the BMJ checklist was the first to emerge and to be used. Published in 1996, it includes 35 items to assess the quality of articles on economic evaluation, and to date this has been the mostly cited of these appraisal methods (20). Each item of the checklist requires a “yes,” “no,” “unclear,” and “not applicable” response, and this broad form of grading means that the checklist may be used to assess various economic evaluations. Due to its wide application, general recognition in the pharmacoeconomic research field, and flexibility of evaluation items, we used BMJ checklist to evaluate the quality of studies included in this review. The percentage of the applicable items on the BMJ checklist meeting by each study represented the quality score of the study.

The 35 checklist items are organized into 10 sections under three headings including study design, data collection, and analysis and interpretation of results. One mark was awarded for each item with a “yes” response, and a mark of zero for “no” or “unclear.” Each study was scored based on its maximum possible score on the checklist, excluding items irrelevant to the study. The overall quality score was calculated as the mean of the scores of all included studies. A score <0.90 was taken to indicate high quality. Scores of individual items were also recorded.

## Results

### Study Description

**Figure 1:**
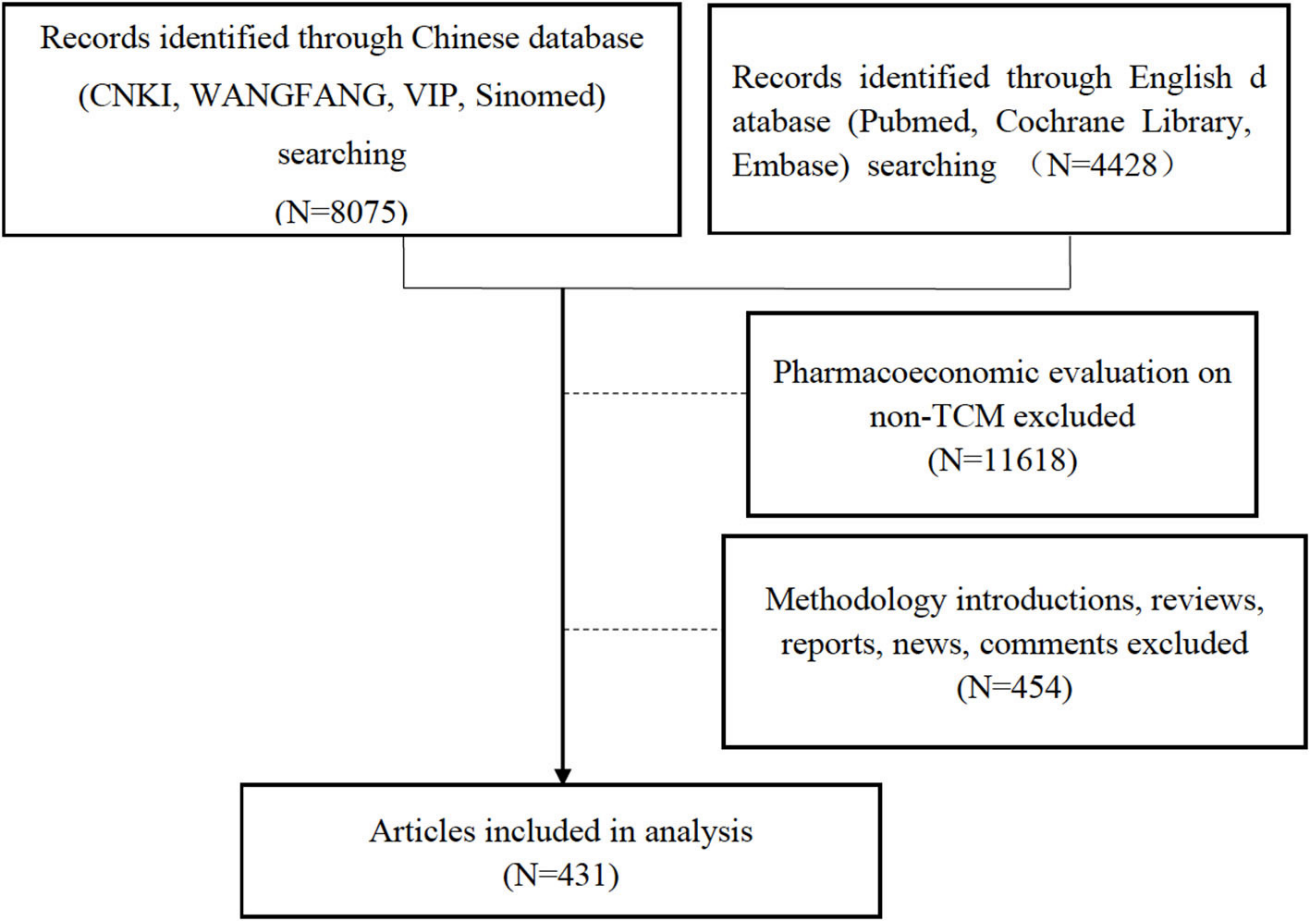
Flow chart of study search and selection.

According to the pre-defined search strategy and selection criteria, the databases were searched and identified articles were screened (Figure 1). We eventually included a total of 431 articles including 424 articles in Chinese language and seven articles in English.

**Figure 2:**
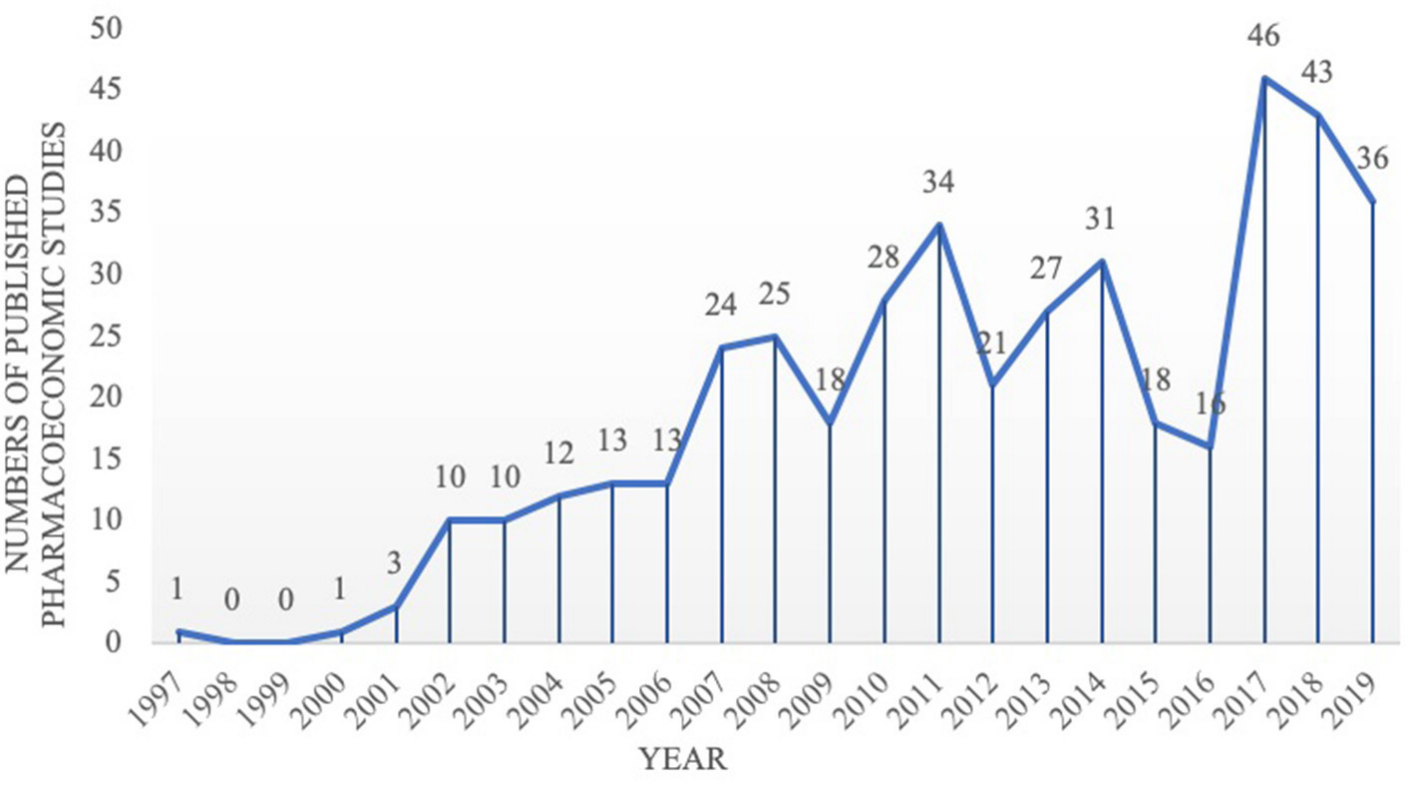
Number of included pharmacoeconomic research publications (*N* = 434).

The first pharmacoeconomic study on TCM in Chinese was published in Chinese Journal of Pharmacoepidemiology in 1997, and focused on Ahylysantinfarctase and Fufang Danshen injection for cerebral infarction patients (21). The first TCM pharmacoeconomic study in English was published in the Chinese Journal of Integrative Medicine in 2014, and was focusing on Chinese medicine and Western medicine for ischemic stroke patients (22). Since 2002, the number of published pharmacoeconomic studies on TCM has gradually increased (Figure 2).

In addition to 25 dissertations, other pharmacoeconomic evaluation literatures on TCM were published in 173 journals, among which 178 were Chinese journals and 5 English journals.

More than 80.97% of the pharmacoeconomic evaluation studies on TCM were carried out by teams with the first author affiliated with a hospital (349), and the remaining studies' first authors were affiliated with universities or research institutions (*n* = 72, 16.71%) or enterprises (*n* = 10, 2.32%). All of the first authors of English articles were affiliated with universities.

Among 431 articles, only 66 (15.56%) were reported as having financial support, including nine nationally funded projects, 22 provincial and ministerial funded projects, 22 municipally funded projects, four university funded projects, four funded projects by the authors' units, three projects supported by a foundation, and two projects supported by Enterprise funding.

### Diseases

**Figure 3:**
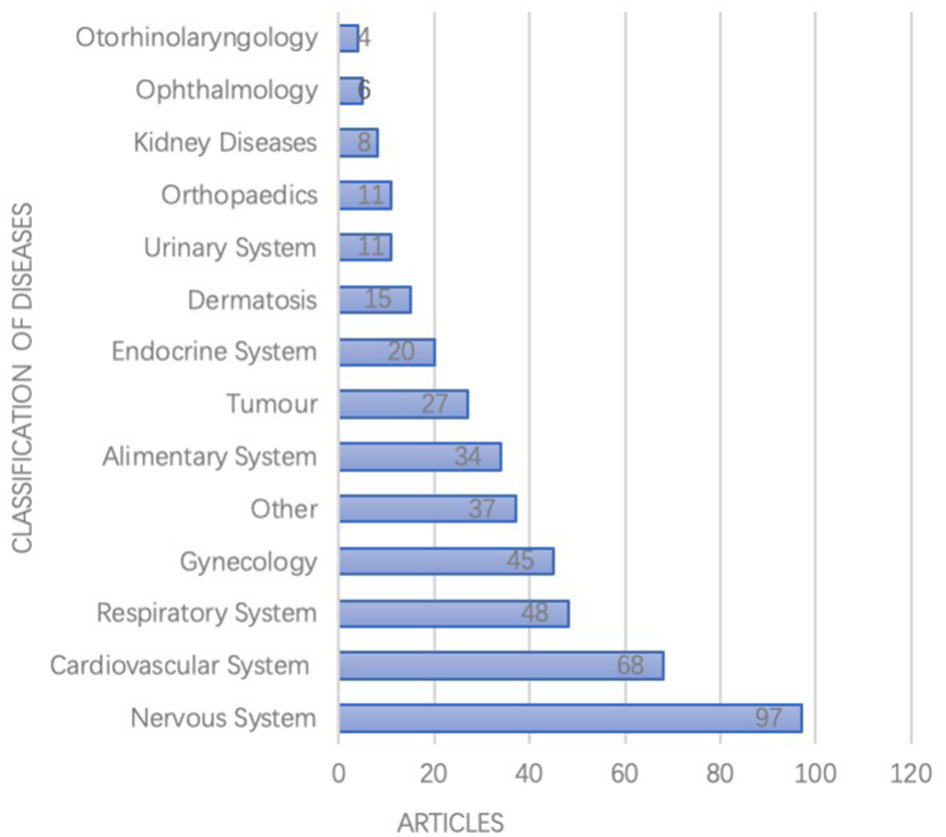
Disease therapies for included pharmacoeconomics researches (*N* = 434).

According to the International Classification of Diseases (ICD-10), the studies focused on a range of 148 kinds of diseases, among which most frequently studied were diseases of the nervous system, cardiovascular system, respiratory system, gynecological, and alimentary systems as shown in Figure 3.

### Interventions

The medical interventions involved in the studies were complex and diverse. Nearly half of the studies compared two interventions, while others compared three or more interventions. However, 186 studies did not describe reasons for choosing the specific program or interventions for comparison. More than a third of the studies used TCM as the control group, and in most of these cases the TCM was not recommended by relevant clinical guidelines (Table 1).

**Table 1:** Number of studies making different types of comparisons between Chinese and Western medicine (*N* = 434).

**Treatment comparison**	**Number of studies**	**Percentage**
Chinese medicine vs. Chinese medicine	164	37.79%
Chinese medicine vs. Western medicine	156	35.94%
Chinese medicine plus western medicine vs. Western medicine	76	17.51%
Chinese medicine plus Western medicine vs. Chinese medicine plus Western medicine	20	4.61%
Chinese medicine prescriptions	15	3.46%
Chinese medicine plus Western medicine vs. Chinese medicine	3	0.7%

The studies included 264 types of Chinese patent medicines and 70 types of TCM prescriptions (such as prescription, pieces decoction, powder, extraction, tincture, and pills). Table 2 shows the Chinese patent medicines that were the focus of more than eight pharmacoeconomic studies, in addition to their TCM syndromes and their indications from the perspective of Western medicine.

**Table 2:** Chinese patent medicines with more than eight pharmacoeconomic studies, the TCM syndromes to which they apply, and the Western medicine indicators for these (*N* = 434).

**Chinese patent medicines**	**Applicable TCM Syndromes**	**Indications of Western Medicine**	**Number of studies**	**Percentage**
Xuesaitong tablet	Blood-activating, stasis-dissolving	Stroke, hemiplegia and others	20	4.60%
Fufang Danshen tablet/dropping pill	Blood-activating, stasis-dissolving	Coronary heart disease, angina pectoris	17	3.92%
Shuxuening injection	Blood vessel-expanding, microcirculation-improving	Ischemic cardiovascular, cerebrovascular diseases, coronary heart disease, angina pectoris, cerebral embolism and others	16	3.69%
Danhong injection	Blood-activating, stasis-dissolving	Chest pain, chest tightness, palpitation, cerebrovascular diseases, coronary heart disease, angina pectoris and others	15	3.46%
Qingkailing injection	Heat clearing-detoxication, tranquilizing,allaying excitement	Upper respiratory tract infection, viral cold and others	15	3.46%
Shuxuetong injection	Blood circulation promoting, blood stasis removing, channels and collaterals dredging	Hemiplegia, speech askew, speech astringent, acute cerebral infarction	13	2.99%
Xiyanping injection	Heat clearing-detoxication, relieve diarrhea	Bronchitis, tonsillitis, bacillary dysentery	13	2.99%
Danshen Chuanxiongqin injection	Not provided	Occlusive cerebrovascular diseases	13	2.99%
Rupixiao tablet	Blood circulation promoting, blood stasis removing, hard mass softening and resolving	Lobular hyperplasia of mammary gland, ovarian cyst, uterine leiomyoma	12	2.76%
Fufang danshen injection	Blood circulation promoting, blood stasis removing, pulse-invigorating, heart-nourishing	Coronary heart disease, chest tightness, angina pectoris	12	2.76%
Ciwujia injection	Liver and kidney reinforcing moderately, bone strengthening	Transient ischemic attack, cerebral arteriosclerosis, cerebral thrombosis and cerebral embolism caused by liver and kidney deficiency, coronary heart disease, angina pectoris, neurasthenia, climacteric syndrome	11	2.53%
Shenmai injection	Qi-supplementing, yin-nourishing, normal pulse restoring, collapse remedy	Palpitation, shortness of breath, limb cold, sweating, pulse loss, myocardial infarction, cardiogenic shock	10	2.30%
Xueshuantong injection	Blood circulation promoting	Central Retinal vein occlusion, sequelae of cerebrovascular disease, internal ophthalmopathy, anterior chamber hemorrhage and others.	10	2.30%
Tanreqing injection	Heat clearing-detoxication, phlegm resolving	Fever, cough, expectoration, sore throat, thirst, red tongue, yellow fur; early pneumonia, acute bronchitis, acute attack of chronic bronchitis, upper respiratory tract infection.	9	2.07%
Yinxingdamo injection	Not provided	Coronary heart disease, thromboembolic diseases	9	2.07%
Yanhuning injection	Not provided	Viral pneumonia, upper respiratory tract infection	9	2.07%
Reduning injection	Heat clearing-detoxication, wind dispelling	High fever, head and body pain, cough, yellow phlegm, upper respiratory tract infection	9	2.07%
Aidi injection	Heat clearing-detoxication, blood stasis eliminating	Primary liver cancer, lung cancer, rectal cancer, malignant lymphoma, gynecological malignancies, and others	8	1.84%

### Study Characteristics

Study characteristics of the 434 studies are presented in Table 3 including the study design, duration, sample size, and viewpoint/perspective. While 24 studies were based on data from previous research, most were prospective or retrospective clinical trials. Seventeen studies did not specify the study duration, but of the remainder most were <3 months. Excluding the 27 studies in which data were extracted from previous research, the sample size of included studies ranged from 38 to 2,820 and subjects' age ranged from 0 to 90 years old. Most (*n* = 377; 86.87%) of the studies did not specify the perspective of their pharmacoeconomic evaluations.

**Table 3:** Characteristics and uncertainty analysis variables for included pharmacoeconomic studies (*N* = 434).

**Study characteristics**	**Number of studies**	**Percentage**
Study design	407	
Prospective study	248	60.93%
Prospective experimental study	234	57.49%
Prospective observational study	14	3.44%
Retrospective study	159	39.07%
Study duration	407	
<14 d	166	40.79%
15 to 30 d	100	24.57%
1 to 3 m	100	24.57%
4 to 6 m	11	2.70%
7 to 12 m	10	2.46%
12 to 24 m	4	0.98%
Not mentioned	16	3.94%
Sample size	407	
<100 subjects	152	37.35%
101 to 200 subjects	146	35.87%
201 to 500 subjects	86	21.13%
501 to 1,000 subjects	15	3.69%
over 1,000 subjects	8	1.97%
Perspective	434	
Medical institutions	24	5.53%
Patients	9	2.07%
Whole society	11	2.53%
Payers	11	2.53%
Health system	2	0.46%
Not specified	377	86.87%
Evaluation Methods	434	
CEA	369	85.02%
CMA	20	4.72%
CBA	7	1.61%
CUA	7	1.61%
Combination analysis	31	7.14%
Uncertainty analysis variables	289	
Drug prices	190	65.74%
Drug prices and other cost (such as examination fee, examination fee, bed fee, treatment fee, nursing fee, compensation/fee for absence from work.)	67	23.18%
Drug prices and health outcome (such as efficiency rate)	19	6.57%
Other cost (such as examination fee, examination fee, bed fee, treatment fee, nursing fee, compensation for one's absence from work)	7	2.42%
Other variables (such as course of medication, discount rate)	6	2.08%

*d, day; m, month*.

More than 85% of the studies used cost-effectiveness analysis (CEA), and the remainder study used at least one method of cost analysis, such as Cost Minimization Analysis (CMA), Cost Benefit Analysis (CBA), or Cost Utility Analysis (CUA). See Table 3 for details. Among these studies, 28 studies used modeling research, with six studies using Markov model and 22 studies using a decision tree model. Thirty-one studies used combination analyses, with two or more methods such as CEA, CMA, CUA, or CBA used in one study. Other relevant specific information is shown in Table 3.

### Cost Scope and Identification

More than 80% of the studies only calculated direct costs, less than one-fifth of the studies included both direct and indirect costs, and the remaining 6% of the studies did not clearly define costs.

Direct costs in these studies were related to the following: drugs (*n* = 385), examination fee (*n* = 158), treatment fee (*n* = 78), hospitalization cost (*n* = 42), nursing cost (*n* = 42), bed fee (*n* = 41), registration fee (*n* = 32), dispensing fee (*n* = 27), adverse reaction treatment cost (*n* = 23), material cost (*n* = 16 studies), meal (*n* = 11), surgery cost (*n* = 9), disposal cost (*n* = 6), consultation fee (*n* = 1), blood transfusion cost (*n* = 1), instrument loss (*n* = 1), and other expenses (*n* = 55). Indirect costs were related to time cost (*n* = 22), compensation (fee) for one's absence from work (*n* = 18), labor loss (*n* = 3), and funeral expenses (*n* = 1).

### Health Outcomes

A total of 226 studies used a single end-point, 156 studies used double end-points, 26 studies used three end-points, and the other 26 studies used four or more end-points at the same time. Of 400 CEA studies with a health-related the final end-points, 264 studies included clinical efficiency/improvement rate, 132 included adverse reactions rate, 47 included efficiency rate of TCM syndromes, 11 included recurrence rate, and 16 included disease or drug withdrawal rate, and 5 included death rate. Fourteen percent (*n* = 61) of studies reported the intermediate end-points as the health-related outcomes, including score/index scale (*n* = 41), biochemical indicators (*n* = 22), physiological indices (*n* = 9), and imaging indices (*n* = 9). Twenty studies reported quality-adjusted life year as a health outcome and one study reported net benefit as a health outcome.

### Uncertainty Analysis and Generalizability

Sixty seven percent of the included studies (*n* = 289) carried out an uncertainty price analysis, the main variables of which are shown in Table 3. In 220 (76.12%) studies, uncertainty analysis were conducted by using one-way sensitivity analysis with reducing drug prices by 10–20%, while 69 (23.88%) of studies used multi-way sensitivity analysis for uncertainty.

A total of 102 articles (23.67%) discussed the limitations of their studies, and 12 articles discussed the generalizability of their results. Although 66 studies included children under 18 years of age, none of these acknowledged the related ethical issues.

### Quality Assessment of Pharmacoeconomic Evaluation Studies on TCM

The average overall quality score of 431 pharmacoeconomics studies on TCM was 0.62.with a range from 0.38 to 0.85. Scores on most of the studies were from 0.5 to 0.7. The distribution is shown in Table 4. On items 2.5, 2.9, 2.12, 3.2, 3.3, 3.4, and 3.5 the score was below 0.1. On items 1.3, 2.7, 2.8, and 3.14 it was no more than 0.5 and on items 1.4, 2.1, 3.6, 3.7, and 3.8 it was no more than 0.7 (Table 4).

**Table 4:** Quality Evaluation Scoring of Included Pharmacoeconomic Studies on TCM (N = 434).

**Evaluation items**		**Scores of the studies**		**Scores of the studies**	
		**published in Chinese**		**published in English**	
		**Number of studies scored**	**Mean score**	**Number of studies scored**	**Mean score**
Study Question	1.1 The research question was stated	427	1	7	1
	1.2 The economic importance of the research question was stated	310	0.726	4	0.571
	1.3 The viewpoint(s) of the analysis were clearly stated and justified	58	0.136	4	0.571
Selection of Alternatives	1.4 The rationale for choosing the alternative programs or interventions compared was stated	241	0.564	3	0.429
	1.5 The alternatives being compared were clearly described	385	0.902	7	1
Form of Evaluation	1.6 The form of economic evaluation used was stated	427	1	7	1
	1.7 The choice of form of economic evaluation was justified in relation to the questions addressed	427	1	7	1
Effectiveness Data	2.1 The source(s) of effectiveness estimates used were stated	214	0.501	6	0.857
	2.2 Details of the design and results of effectiveness study were given (if based on a single study)	253	0.811	7	1
	2.3 Details of the method of synthesis or meta-analysis of estimates were given (if based on an overview of a number of effectiveness studies)	22	0.880	0	0
Benefit Measurement and Valuation	2.4 The primary outcome measure(s) for the economic evaluation were clearly stated	427	1	7	1
	2.5 Methods to value health states and other benefits were stated	0	0	1	0.143
	2.6 Details of the subjects from whom valuations were obtained were given	392	0.918	6	0.857
Costing	2.7 Productivity changes (if included) were reported separately**Δ**	44	0.103	0	0
	2.8 The relevance of productivity changes to the study question was discussed**Δ**	57	0.133	0	0
	2.9 Quantities of resources were reported separately from their unit costs**Δ**	0	0	2	0.286
	2.10 Methods for the estimation of quantities and unit costs were described	402	0.941	6	0.857
	2.11 Currency and price data were recorded	417	0.977	5	0.714
	2.12 Details of currency of price adjustments for inflation or currency conversion were given	0	0	0	0
Modeling	2.13 Details of any model used were given	20	0.833	4	1
	2.14 The choice of model used and the key parameters on which it was based were justified	22	0.917	4	1
Adjustments for timing of costs and benefits	3.1 Time horizon of costs and benefits was stated	433	1	6	0.857
	3.2 The discount rate(s) was stated^*^Δ	7	0.016	3	0.429
	3.3 The choice of rate(s) was justified^*^Δ	7	0.016	3	0.429
	3.4 An explanation is given if costs or benefits were not discounted^*^Δ	17	0.040	1	0.143
Allowance for uncertainty	3.5 Details of statistical tests and confidence intervals were given for stochastic data^*^	25	0.059	4	0.571
	3.6 The approach to sensitivity analysis was given	284	0.665	5	0.714
	3.7 The choice of variables for sensitivity analysis was justified	285	0.667	4	0.571
	3.8 The ranges over which the variables were varied are stated	283	0.663	5	0.714
	3.9 Relevant alternatives were compared	427	1	7	1
Presentation of results	3.10 Incremental analysis was reported	267	0.837	6	0.857
	3.11 Major outcomes are presented in a disaggregated as well as aggregated form	427	1	7	1
	3.12 The answer to the study question was given	427	1	7	1
	3.13 Conclusions followed from the data reported	427	1	7	1
	3.14 Conclusions were accompanied by the appropriate caveats^*^	77	0.180	7	1

* *indicates that the score for studies in English was higher than in Chinese; Δ indicates that the score of studies was relatively low both in Chinese and English articles*.

The quality of the studies was not significantly improved over the past 20 years, as shown in Table 5. The quality of studies with first authors affiliated with universities researchers was much higher than that of studies with first authors affiliated with hospitals. The mean score of the seven studies in English was 0.72, while that of studies published in Chinese journals was 0.62. The scores of studies in English were higher than those in Chinese on items 3.2, 3.2, 3.4 3.5, and 3.14 and scores were relatively high in the Chinese journals on items 1.2, 2.3, 2.7, and 2.8. However, scores on items 2.7, 2.8, 2.9, 3.2, 3.3, and 3.4 were low in both Chinese and English studies, suggesting a need for improvement (Table 4).

**Table 5:** Scoring comparison from different viewpoints for included pharmacoeconomic studies (*N* = 431).

**Viewpoint**	**Number of studies**	**Score**
**Year of publication**		
Before 2005	50	0.62
2005–2010	108	0.62
2011–2015	132	0.61
After 2015 y	144	0.63
**Language of publication**		
Studies in Chinese	427	0.62
Studies in English	7	0.72
**First author affiliation**		
Hospital	349	0.61
University	75	0.68
Enterprise	10	0.64

## Discussions

Policy orientation and a demand for evidence have influenced a rapid increase over the past decade in number of the published pharmacoeconomic research articles on TCM. However, the volume remains only one tenth of the number of corresponding articles on chemical drugs. Overall, the quality of published pharmacoeconomics evaluation on TCM was relatively and generally low, which was consistent with the results of several studies in 2009 (23), 2015 (24), and 2020 (25). And compared with the studies of Chen (26), Li (27), Lei (28) in 2004, 2008, and 2010 respectively, the quality of pharmacoeconomics research articles on TCM was also lower than that on chemical drugs.

Three main limitations in quality were identified. Firstly, many of the included studies were not well-designed and lacked clear research perspective. Only four studies described the method of sample size estimation based on China guidelines for pharmacoeconomic evaluation, which would affect cost identification and measurement and thereby the integrity and reliability of the studies (29). Many studies did not report the randomization method, and failed to justify the selected intervention.

Many studies with first authors affiliated with a hospital appeared to not fully understand the basic principles of pharmacoeconomics and did not design their clinical trials appropriately, despite pharmacoeconomic data being relatively easy to obtain in a hospital. Some studies failed to implement and apply pharmacoeconomic research methods correctly. There were limitations or errors in cost identification and measurement in some studies, more than half of which included only drug costs. Due to these issues, some researchers misunderstood or were confused about cost assessment in pharmacoeconomic evaluation. Even in the study of chronic diseases, people generally did not pay enough attention to discount. In addition, the choice of discount rate is also based on different countries and different perspectives, which is also a controversial issue in the field of Pharmacoeconomics.

Some studies assessed TCM as an adjunct to Western medicines, which made the cost difficult to calculate. In addition, the lack of a specific and unique pharmacoeconomic evaluation guideline for TCM was also an important limitation. In 2013, Xie et al. published a “technical specification for pharmacoeconomic evaluation on post marketed Chinese patent medicine (Draft)” in the China Journal of traditional Chinese Medicine (30). This guideline considered the basic ideology and characteristics of traditional Chinese medicine to a certain extent, but did not solve the specific problems in the pharmacoeconomic evaluation of TCM, such as how to map the effectiveness and health outcome of TCM and chemical medicines.

Finally, some TCM clinical trials were found to be limited in terms of sample size, comparison type, or the use of non-scientific assessments. The short durations of most studies were not well-aligned with the chronic nature of disease on which they focused, and did not fully reflect the advantages of TCM in improving long-term clinical efficacy and potential safety. Most of the studies were retrospective, with small samples and no follow-up. Common defects were in protocol design, lack of trial registration, study reporting, and quality control. Frequently used outcome indicators were overall efficiency and recurrence rate, and were not sufficiently specific to reflect outcomes of different types of TCM treatment.

There were some limitations in our study. First, the BMJ checklist was published in the 1990s, and its applicability may deviate to some extent from the current research including pharmacoeconomic evaluations on TCM. Second, this study included only published research and not unpublished research reports. Some outcome indicators were not addressed by the included studies, resulting in low scores for those indicators, which may have impacted negatively on the evaluation results of this study.

## Conclusions

Although limitations and deficiencies are found in the current pharmacoeconomic evaluation of TCM, the body of research still provides a lot of valuable evidence for the rational use of health resources, essential medicine and state medical insurance catalog selection and national drug price negotiations. The following recommendations may improve the quality of research in this area. In the pharmacoeconomics evaluation of TCM, the following recommendations are made.

With regard to the point discussed above, we call for enhanced methodological training and cooperation to improve the quality of research and reporting quality. A pharmacoeconomic evaluation guideline conforming to the theoretical characteristics of TCM should be established to reflect the economic evaluation results of TCM objectively. In addition, importantly, a reasonable health outcome index for TCM should be developed, balancing between the specificity and quantification of TCM indicators, mapping of indicators between Chinese patent medicine, and Western medicines, and enhancing clinical comparability of health indicators.

## Data Availability Statement

The original contributions presented in the study are included in the article/supplementary material, further inquiries can be directed to the corresponding author/s.

## Author Contributions

MH and NY conceived the study design. HZ and TD searched and selected the articles, extracted, analyzed, and interpreted the data. NY drafted the manuscript. MH and JG critically reviewed the manuscript. All authors read and approved the final version of the manuscript.

## Conflict of Interest

The authors declare that the research was conducted in the absence of any commercial or financial relationships that could be construed as a potential conflict of interest.

## Publisher's Note

All claims expressed in this article are solely those of the authors and do not necessarily represent those of their affiliated organizations, or those of the publisher, the editors and the reviewers. Any product that may be evaluated in this article, or claim that may be made by its manufacturer, is not guaranteed or endorsed by the publisher.

## Abbreviations

CAM: complementary and alternative medicine
TCM: traditional Chinese medicine
PRISMA: preferred reporting items for systematic reviews and meta-analyses
CNKI: China national knowledge infrastructure
VIP: Chongqing VIP information
BMJ: British medical journal
QHES: quality of health economics studies
CHEC: consensus on health economic criteria
ISPOR: international society of pharmacoeconomics and outputs research
CHEERS: consolidated health economic evaluation reporting standards
CEA: cost-effectiveness analysis
CMA: cost analysis
CBA: cost-benefit analysis
CUA: cost-effectiveness analysis
DT: decision tree.
